# Mental health culturomics

**DOI:** 10.1371/journal.pmen.0000135

**Published:** 2024-09-11

**Authors:** Ivan Jarić, Joseph Firth, Uri Roll, Josh A. Firth

**Affiliations:** 1 Université Paris-Saclay, CNRS, AgroParisTech, Ecologie Systématique Evolution, Gif-sur-Yvette, France; 2 Institute of Hydrobiology, Biology Centre of the Czech Academy of Sciences, České Budějovice, Czech Republic; 3 Division of Psychology and Mental Health, University of Manchester, Manchester Academic Health Science Centre, Manchester, United Kingdom; 4 Greater Manchester Mental Health NHS Foundation Trust, Manchester Academic Health Science Centre, Manchester, United Kingdom; 5 Mitrani Department of Desert Ecology, The Jacob Blaustein Institutes for Desert Research Ben-Gurion University of the Negev, Midreshet Ben-Gurion, Israel; 6 School of Biology, University of Leeds, Leeds, United Kingdom; 7 Department of Biology, University of Oxford, Oxford, United Kingdom; PLOS: Public Library of Science, UNITED KINGDOM OF GREAT BRITAIN AND NORTHERN IRELAND

The ongoing digital revolution is giving rise to an increasing amount of online data regarding health-related issues. Specifically, mental health issues have a particularly high and increasing amount of dedicated online search activity and online discussions through social media. These data represent a valuable source on societal awareness, knowledge, interests, and attitudes, regarding the prevalence of mental health conditions across space and time [1]. Nevertheless, despite this exceptional digital footprint, a standardised toolkit and ethical framework to investigate mental health data and insights using online digital sources is largely lacking.

Specifically, digital technologies and ubiquitous internet use in daily human activities are opening novel research avenues across sciences [1]. They are encapsulated by an emerging field of ‘culturomics’, which aims to study human behaviour and cultural dynamics through the quantitative analysis of the ever-growing digital data corpora [2,3]. Here, we propose that combining the framework of culturomics with the wealth of online activity surrounding mental health can be used to create a cohesive research area named ‘mental health culturomics’. Culturomic data are diverse (Fig 1) and include, for example, search engine patterns, social media posts and engagement, online news articles, online encyclopaedia content and viewership, and images and videos from media sharing platforms [3,4]. The analysis of these data through various, often advanced analytical methods, can provide information such as spatiotemporal distribution and trends of studied phenomena and processes, public sentiments, topic popularity, and societal network functioning [3,4]. Culturomic data can generate novel insights at a high spatio-temporal resolution, such as understanding human behavioural and cognitive patterns, daily routines and rhythms, attention, interests, attitudes, societal norms, and cultural values [1]. While culturomics is increasingly being applied across a wide range of disciplines including social sciences, the humanities, and various STEM fields [3–5], the concept ‘mental health culturomics’ has yet to be properly defined, applied or critiqued, despite increasing number of studies over the past decade.

**Fig 1:**
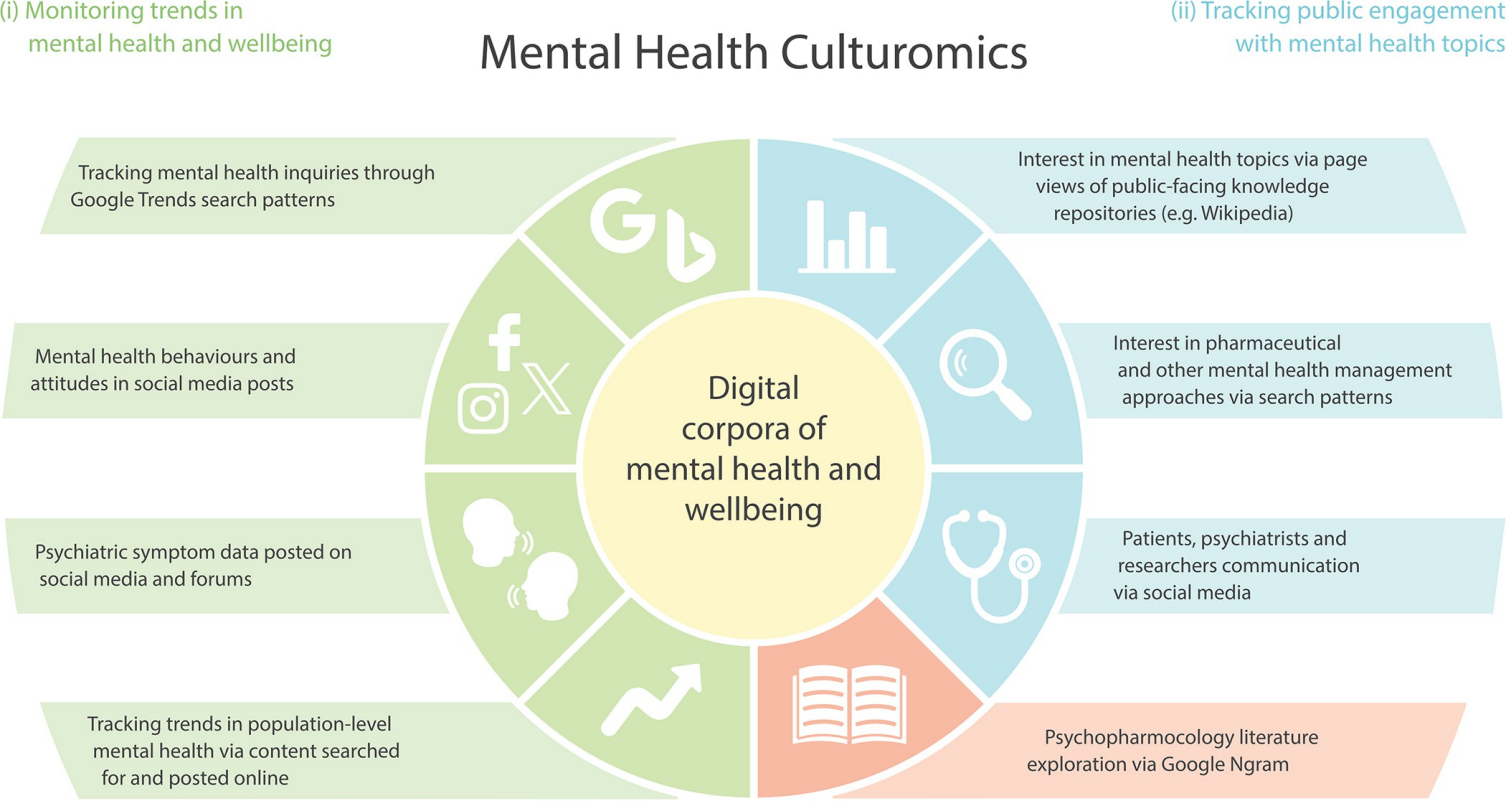
Conceptual representation of mental health culturomics with key applications.

Given the amount of online activity in the area of mental health, culturomics holds many possible applications, including monitoring treatment outcome reporting, discovering trends in public behavioural mental health habits and lifestyles, tracking access to healthcare services, along with improving the understanding of online discourse surrounding mental health-related issues [1]. For example, a study of the language features used to communicate self-reported depression on the Facebook social media platform identified significant race-based differences [6], while an assessment of depression-related health online information seeking (i.e., using Google Trends platform) revealed clear diurnal patterns in depression-related interest [7]. A study of the global prevalence of biophobias, negative responses to certain natural stimuli associated with severe psychological problems such as anxiety and panic attacks, revealed a steady increase in global public interest in biophobias worldwide based on online search patterns [8]. A global analysis of millions of Twitter posts revealed independent diurnal and seasonal variation of positive and negative affects (i.e., enthusiasm and delight vs. fear and anger), as well as their association in seasonal peaks in depression anxiety [9]. In another study, Twitter posts and YouTube videos were assessed to identify societal shifts in circadian rhythms towards nocturnal activity and the levels of emotional resilience during COVID-19 lockdowns [10]. A general framework of ‘mental health culturomics’ could bring such efforts together in a unified manner that will contribute to sharing and developing approaches, tools, data, challenges, and how to address them. In particular, we propose that the diverse applications of ‘mental health culturomics’ can be classified into two main categories: (i) monitoring mental health and wellbeing trends in space and time such as prevalence or spread, and (ii) investigating public engagement with mental health-related topics, encompassing attitudes, values, awareness levels, and interest dynamics (Fig 1).

In sum, due to the exceptional relationship between mental health and the online world, culturomics has a strong potential to provide critical insights into the complex interplay between societal dynamics and mental health-related phenomena, and provide valuable and inexpensive data. While the field of culturomics and its scientific community have so far applied these concepts and methods mostly outside the context of mental health, their integration and better recognition within the field of mental health science is now possible and urgently needed. This will enable currently-disparate methods and approaches within this topic to be joined under a single and established framework, as well as allowing the potential for these investigations to become more widely recognised, ethical issues to be addressed at scale, and standardised processes for data analysis and interpretation to occur. We propose that future developments in regard to formalising ‘mental health culturomics’ as a field and advancing the applications are likely to be transformative for the field, with a potential to catalyse a paradigm shift in research methodologies, and bring forward new insights and innovation.

## References

[1] Firth J, Torous J, López-Gil JF, Linardon J, Milton A, Lambert J, et al. From “online brains” to “online lives”: understanding the individualized impacts of Internet use across psychological, cognitive and social dimensions. World Psychiatry. 2024;23:176–90. doi: 10.1002/wps.21188 38727074 PMC11083903

[2] Michel JB, Shen YK, Aiden AP, Veres A, Gray MK, et al. Quantitative analysis of culture using millions of digitized books. Science. 2011;331:176–82. doi: 10.1126/science.1199644 21163965 PMC3279742

[3] Correia RA, Ladle R, Jarić I, Malhado ACM, Mittermeier JC, Roll U, et al. Digital data sources and methods for conservation culturomics. Conserv Biol. 2021;35:398–411. doi: 10.1111/cobi.13706 33749027

[4] Jarić I, Correia RA, Brook BW, Buettel JC, Courchamp F, Di Minin E, et al. iEcology: harnessing large online resources to generate ecological insights. Trends Ecol Evol. 2020;35:630–39. doi: 10.1016/j.tree.2020.03.003 32521246

[5] Lazer D, Hargittai E, Freelon D, Gonzalez-Bailon S, Munger K, Ognyanova K, et al. Meaningful measures of human society in the twenty-first century. Nature. 2021;595(7866):189–196. doi: 10.1038/s41586-021-03660-7 34194043

[6] Rai S, Stade EC, Giorgi S, Francisco A, Ungar LH, Curtis B, et al. Key language markers of depression on social media depend on race. Proc Natl Acad Sci USA. 2024;121:e2319837121. doi: 10.1073/pnas.2319837121 38530887 PMC10998627

[7] Tana JC, Kettunen J, Eirola E, Paakkonen H. Diurnal variations of depression-related health information seeking: case study in Finland using Google Trends data. JMIR Ment Health. 2018;5(2):e9152. doi: 10.2196/mental.9152 29792291 PMC5990858

[8] Correia RA, Mammola S. The searchscape of fear: A global analysis of internet search trends for biophobias. People Nat. 2023;6(3):958–972.

[9] Golder SA, Macy MW. Diurnal and seasonal mood vary with work, sleep, and daylength across diverse cultures. Science. 2011;333(6051):1878–1881. doi: 10.1126/science.1202775 21960633

[10] Castaldo M, Venturini T, Frasca P, Gargiulo F. The rhythms of the night: increase in online night activity and emotional resilience during the spring 2020 Covid-19 lockdown. EPJ Data Sci. 2021;10(1):7. doi: 10.1140/epjds/s13688-021-00262-1 33552837 PMC7848867

